# P-492. Quantification of HIV Shedding in Viremic Patients

**DOI:** 10.1093/ofid/ofae631.691

**Published:** 2025-01-29

**Authors:** Rebecca Linfield, Alessandro Zulli, Sehee Jong, Marlene Wolfe, Alexandria Boehm, Julie Parsonnet

**Affiliations:** Stanford University, Stanford, California; Stanford University, Stanford, California; Stanford University, Stanford, California; Emory University; Stanford University, Stanford, California; Stanford School of Medicine, Stanford, California

## Abstract

**Background:**

Identifying pockets of undiagnosed HIV is a critical step in the U.S. Ending the HIV Epidemic (EHE). Wastewater-based epidemiology represents a novel way to build agnostic population-level data about disease distribution. We detected HIV virus in two sewersheds in Northern California. We are now conducting a study to measure how much HIV virus is shed into urine and stool, to better estimate cases represented by wastewater detection.

HIV-1 RNA Detected in Concentrated Urine

**Figure:**
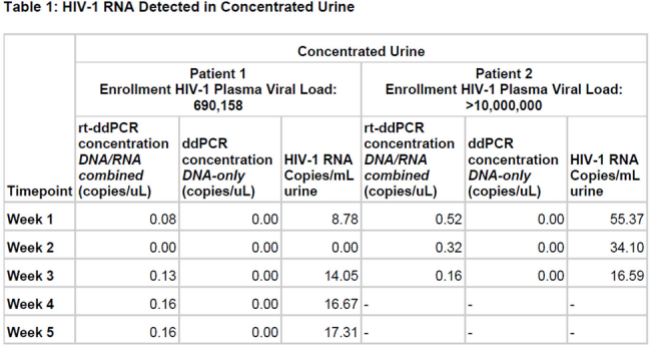

**Methods:**

We are recruiting participants from HIV clinics in San Mateo and Santa Clara Counties. Patients must be 18 years or older with HIV-1 viral load of >10,000 copies/ml at the time of recruitment. Subjects are asked to provide 5 weekly self-collected blood (Tasso), urine, and stool samples. The plasma is analyzed by established HIV-1 RNA quantitative PCR techniques. Nucleic acids from urine, concentrated (centrifuged) urine, and stool are suspended in DNA/RNA Shield, then extracted using the ZymoBIOMICS DNA/RNA Miniprep Kit (ZymoBIOMICS #R2002). HIV genome-specific primers target the Long Terminal Repeat (LTR) region of the HIV-1 genome. Droplets for digital droplet PCR are generated using the AutoDG Automated Droplet Generator. Reverse transcriptase PCR, which quantifies RNA and DNA combined, and direct PCR, which quantifies DNA, is then carried out with the Mastercycler Pro. Thresholding is performed using QX Manager Software.

HIV-1 RNA Detected in Stool

**Figure:**
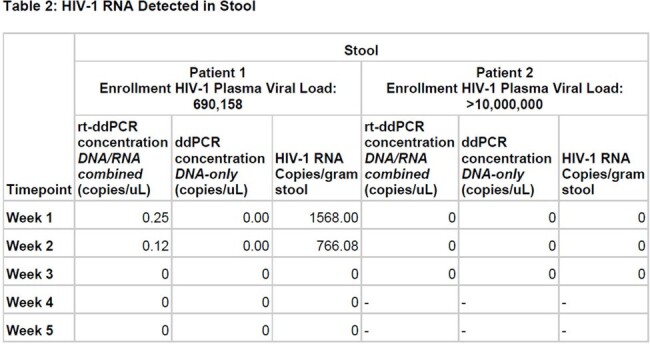

**Results:**

Two patients have been enrolled to date. Patient 1 was started on antiretroviral therapy after the first sample was obtained; Patient 2 was started on antiretroviral therapy 10 days before the first sample was collected. HIV-1 RNA was found in 87.5% (7/8) of concentrated urine samples, including 85.7% (6/7) of samples obtained while on ART (Table 1). Among stool samples, 25.0% (2/8) yielded HIV RNA, but only 14.3% (1/7) of those obtained on ART (Table 2). Nucleic acids were not detected in unconcentrated urine. Plasma viral load testing is pending, so only the initial clinical HIV-1 viral loads at enrollment are shown.

**Conclusion:**

HIV-1 RNA is present in concentrated urine but also in stool samples of viremic patients, even up to five weeks after initiation of ART. When the study is complete, our results will bolster how to interpret HIV-1 detected in wastewater.

**Disclosures:**

**All Authors**: No reported disclosures

